# Redox–Mitochondria–Immune Network Dysregulation in Schizophrenia: From Selective Cellular Vulnerability to Circuit Dysfunction

**DOI:** 10.3390/cells15131153

**Published:** 2026-06-25

**Authors:** Tingyan He, An Yu, Yulin Qian, Tonglin Wu, Changguo Ma

**Affiliations:** Yunnan Key Laboratory of Cell Therapy for Refractory Diseases, Kunming University, Kunming 650214, China

**Keywords:** schizophrenia, redox dysregulation, mitochondrial dysfunction, neuroinflammation, parvalbumin interneurons, biomarker stratification

## Abstract

**Highlights:**

**What are the main findings?**

- Redox dysregulation in schizophrenia is best viewed as a multiscale redox–mitochondria–immune framework, and this review provides an evidence-weighted multiscale synthesis of a redox–mitochondria–immune network and explicitly separates well-supported links from candidate mechanisms.
- Redox vulnerability is plausibly enriched in parvalbumin-expressing interneurons and oligodendrocyte lineage cells, while astrocytes and pyramidal neurons remain important contextual partners in glutathione supply, excitation–inhibition balance, and circuit dysfunction.

**What are the implications of the main findings?**

- The translational contribution of this review is an evidence-weighted, stage-sensitive stratification framework that separates established findings from animal-model-supported and extrapolated mechanisms.
- We extend the framework by incorporating nitrosative stress and the GPx4–lipid peroxidation–ferroptosis axis as candidate amplification pathways and outline testable predictions for future validation.

**Abstract:**

Oxidative stress has been recognized as a repeatedly validated pathophysiological factor in schizophrenia, but its mechanistic role and translational relevance remain incompletely defined. Prior work has advanced redox dysregulation, neuroinflammation, and NMDA receptor hypofunction as a putative central hub in schizophrenia. This narrative review proposes an evidence-weighted redox–mitochondria–immune framework that integrates peripheral biomarkers, magnetic resonance spectroscopy, postmortem findings, and preclinical mechanisms while explicitly distinguishing established observations from candidate pathways. Existing studies support increased oxidative damage and altered antioxidant buffering in schizophrenia, particularly involving the glutathione system. However, these abnormalities are neither uniform across disease stages nor equally represented across patient subgroups, and may be markedly prominent only in certain biological subgroups. Mechanistically, redox imbalance may interact with mitochondrial bioenergetic deficits and innate immune signaling; however, pathway-specific links such as cGAS-STING activation, nitrosative/peroxynitrite stress, and GPx4-ferroptosis should currently be treated as testable extensions rather than validated human mechanisms in schizophrenia. Importantly, the pathological consequences of oxidative stress are unlikely to be cell-type neutral. Parvalbumin-positive interneurons and oligodendrocyte lineage cells are more vulnerable because of their high metabolic load, limited antioxidant buffering capacity, and lipid/iron-related susceptibility, thereby providing a mechanistic bridge to excitation–inhibition imbalance, myelin abnormalities, and reduced circuit synchrony. Microglial redox–inflammatory signaling may further exacerbate these processes. On the basis of this framework, we argue that the key for future research is not to continue demonstrating the universality of oxidative stress, but to improve the translational efficiency. Biomarker-guided stratification, stage-sensitive study designs, and cell-type-informed therapeutic strategies may therefore provide a more productive path toward redox-targeted interventions in schizophrenia.

## 1. Introduction

Schizophrenia is a severe mental disorder, with main symptoms including positive symptoms, negative symptoms, and cognitive impairment. The disease affects 1% of the world’s population and imposes a heavy burden on society [1]. Because its pathophysiological mechanisms have not yet been fully elucidated, this has limited the development of treatment strategies, especially for cognitive deficits and persistent negative symptoms [2], which currently still cannot be adequately addressed by antipsychotic medications.

In the biological processes involved in schizophrenia, oxidative stress has been regarded as an important pathogenic mechanism leading to disease susceptibility and progression. Numerous studies have documented increased oxidative burden, elevated lipid peroxidation, and impaired antioxidant defenses in patients [3,4], and these changes are associated with symptom severity and cognitive dysfunction. These findings suggest that redox dysregulation is not merely an epiphenomenon but may be integrated into the pathophysiological mechanisms that shape the disease’s manifestations at different stages.

Oxidative stress in schizophrenia appears to play a role within a broader pathological network that includes mitochondrial dysfunction, immune activation, and impaired antioxidant capacity [5,6]. This network has a positive feedback property: mitochondrial abnormalities increase ROS generation, ROS triggers immune activation, and inflammatory signals further exacerbate oxidative damage. Importantly, this redox dysregulation is unlikely to affect all neural cells uniformly. A growing body of evidence indicates that specific cell populations, particularly parvalbumin-positive interneurons and oligodendrocytes, are especially sensitive to oxidative stress [7,8], thereby linking molecular-level oxidative damage to system-level dysfunctions such as excitation–inhibition imbalance and white matter abnormalities. Meanwhile, emerging evidence suggests that redox abnormalities may differ across disease stages and clinical phenotypes [9,10], indicating that oxidative stress not only contributes to core pathophysiological processes but may also explain the clinical heterogeneity of schizophrenia. This perspective helps explain why the clinical effects of antioxidant interventions are inconsistent and why precision treatment strategies based on patient stratification are needed.

The conceptualization of redox dysregulation, neuroinflammation and NMDA-receptor hypofunction as a self-reinforcing central hub, together with the feed-forward vicious-circle model of oxidative stress in schizophrenia, was advanced primarily by Do, Cuénod, Steullet and colleagues [7,11,12]. The aim of this review is therefore not to re-present that framework as new. Instead, we focus on three incremental contributions: first, introduce an explicitly translational, stratification-oriented perspective that addresses stage-dependent vulnerability, biomarker enrichment strategies, target engagement validation, and proximal mechanistic outcome measures; second, systematically map the network according to strength of evidence, distinguishing causal pathways supported by robust data from mechanistic hypotheses that remain tentative; and third, extend this framework to include nitrosative stress and the GPx4–lipid peroxidation–ferroptosis axis, treating these pathways as testable candidate amplifiers with clear, falsifiable predictions.

Accordingly, this review first summarizes evidence supporting redox imbalance in schizophrenia, then examines how oxidative stress may interact with mitochondrial dysfunction and innate immune activation, why certain neural cell populations may be selectively vulnerable, and how these disturbances may propagate to circuit dysfunction and clinical heterogeneity. Finally, we discuss the implications of this framework for stratified and biomarker-informed therapeutic strategies, while highlighting where direct human evidence remains insufficient.

### Literature Identification and Evidence Appraisal

This is a narrative review with an explicit aim of differentiating evidence strength across biomarkers and mechanistic links. We searched PubMed and Web of Science (up to March 2026), combining terms for schizophrenia/psychosis with oxidative stress, redox, glutathione, mitochondria, neuroinflammation, parvalbumin, oligodendrocyte, ferroptosis and the relevant antioxidant compounds, prioritizing meta-analyses and replicated human studies and supplementing them with mechanistic animal and cellular work where human data were sparse.

Because the argument of this review rests on weighing the evidence rather than merely cataloguing it, we apply throughout a clear and intentionally simplified scheme for grading the strength of evidence for each core claim. Established (E) denotes support from replicable human studies and/or meta-analyses; Model-supported (M) indicates consistent preclinical evidence with limited or indirect human validation; Extrapolated (X) represents biological plausibility based primarily on inferences from other diseases or in vitro systems, yet to be directly confirmed in schizophrenia. These gradations are individually annotated in the main text and summarized in Table 1. We adopt this transparent grading scheme rather than formal evaluation tools such as GRADE, as the latter are not suitable for mechanistic narrative synthesis; this grading aims to provide readers with intuitive guidance on evidence confidence rather than quantitative scoring.

## 2. Brain Redox Biology and Antioxidant Systems

**Figure 1:**
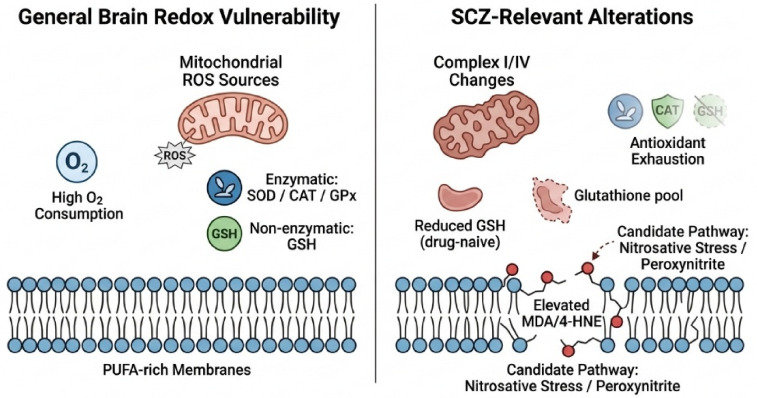
Brain redox vulnerability and schizophrenia-relevant alterations. **Left**: general factors that render the brain intrinsically susceptible to oxidative stress, including high oxygen consumption, polyunsaturated fatty acid-rich membranes, mitochondrial reactive oxygen species production, and relatively limited antioxidant reserve. **Right**: schizophrenia-associated alterations mapped onto this vulnerability framework. Mitochondrial complex I/IV changes have been reported. Enzymatic antioxidant systems show heterogeneous findings consistent with dynamic compensation-to-exhaustion transitions. The glutathione axis shows reduced levels in drug-naïve first-episode schizophrenia, with brain glutathione changes that may be stage-dependent. Lipid peroxidation markers (malondialdehyde, 4-hydroxynonenal) are among the most consistently elevated peripheral indices. Nitrosative stress and peroxynitrite-mediated damage represent candidate amplification pathways for which direct human evidence in schizophrenia remains limited.

Brain redox homeostasis depends on a precise balance between the generation of ROS and their removal by antioxidant systems. Under physiological conditions, ROS act as signaling molecules involved in processes such as synaptic plasticity, learning, and memory [22]. When ROS production exceeds antioxidant defense capacity, oxidative stress is triggered, causing damage to lipids, proteins, and nucleic acids, thereby impairing cellular structure and function (Figure 1).

### 2.1. Why the Brain Is Highly Vulnerable to Oxidative Stress

Brain tissue is particularly sensitive to oxidative damage, mainly due to its high oxygen consumption [23], intensive mitochondrial activity, abundance of unsaturated fatty acids, and enrichment in redox-active metals such as iron and copper [24]. In addition, the antioxidant reserves of neural tissue are relatively limited and difficult to match its vigorous metabolic demands. Together, these characteristics make the brain a high-risk organ for redox imbalance.

### 2.2. Sources of Reactive Oxygen Species in the Brain

Brain ROS are mainly produced through two pathways: first, electron leakage during mitochondrial oxidative phosphorylation; second, enzymatic reactions such as those catalyzed by NADPH oxidase and xanthine oxidase [25]. Because neurons require a continuous energy supply to maintain electrical activity and synaptic transmission, mitochondria-derived ROS are particularly prominent. In addition, the iron-dependent Fenton reaction can generate highly reactive hydroxyl radicals [26]. Under pathological conditions, glutamate excitotoxicity induces calcium overload, aggravates mitochondrial dysfunction, and further amplifies oxidative stress [27].

### 2.3. Antioxidant Defense Systems in the Brain

To maintain redox balance, the brain is equipped with two types of antioxidant systems: enzymatic and non-enzymatic. The enzymatic system includes superoxide dismutase, catalase, and glutathione peroxidase, which sequentially detoxify superoxide and hydrogen peroxide, limiting the generation of more destructive free radicals. Among them, glutathione peroxidase plays a key role in protecting membrane lipids from peroxidation [28]. The non-enzymatic system centers on glutathione, the most abundant intracellular antioxidant in the brain, which not only directly scavenges ROS but also serves as a substrate for multiple antioxidant reactions.

### 2.4. Nitrosative Stress and Oxidative–Nitration Coupling

In addition to ROS, reactive nitrogen species (RNS) are also an important component of the brain’s redox network. Nitric oxide (NO) and its derivatives can react with superoxide anions to form peroxynitrite [29], thereby triggering protein nitration and damage to the mitochondrial respiratory chain. Together with ROS, this process constitutes a mutually amplifying oxidation–nitration coupling mechanism. Because protein nitration can affect the activity of receptors, transporters, and metabolic enzymes [30], RNS-related changes are not only markers of oxidative damage but may also participate in regulating the thresholds of synaptic transmission and inflammatory responses [31]. Incorporating RNS into the framework of redox homeostasis helps explain why, in some studies where no obvious abnormalities are found in certain ROS indicators, functional changes associated with redox imbalance can still be observed, and it also reveals differences among antioxidant treatment strategies at the level of their targets.

Taken together, brain redox homeostasis has intrinsic vulnerability. This vulnerability is highly associated with schizophrenia: persistent redox imbalance can act in concert with mitochondrial dysfunction and immune activation, leading to damage in specific cell types and downstream neural circuit dysregulation.

## 3. Evidence of Redox Dysregulation in Schizophrenia

Overall, studies of peripheral blood, cerebrospinal fluid, magnetic resonance spectroscopy, and postmortem brain tissue support an association between schizophrenia and disrupted redox homeostasis, manifested as increased markers of oxidative damage and abnormalities in antioxidant buffering/defense systems. However, the existing evidence does not point to a single, unified oxidative-stress phenotype; instead, it shows heterogeneity: correlations across indicators in different compartments are limited, and the same indicator may show inconsistent directions or even compensatory changes across different stages of illness, or under different medication exposures and metabolic backgrounds. Therefore, the key to understanding this field lies not only in cataloging abnormalities, but in answering three questions crucial for subsequent mechanistic frameworks: which findings are most reproducible across studies? What are the main systematic sources of heterogeneity? Which evidence is closer to central mechanisms, versus more likely to reflect peripheral comorbidities and lifestyle burdens?

### 3.1. Peripheral Evidence

#### 3.1.1. Oxidative Damage Markers

Among the more repeatedly reported peripheral findings are lipid peroxidation-related markers, although their interpretation depends importantly on assay methodology. Multiple independent studies and meta-analyses have repeatedly observed elevated levels of malondialdehyde (MDA) and 4-hydroxynonenal (4-HNE) in plasma or erythrocytes, suggesting increased free radical-mediated membrane lipid damage and serving as one of the peripheral biological markers of “increased oxidative burden” [13,14]. Protein oxidation markers such as advanced oxidation protein products (AOPPs) have also shown an upward trend in some studies, further supporting the presence of a systemic oxidative stress background in patients with schizophrenia [16].

However, the interpretation of MDA requires methodological caution. In schizophrenia studies, MDA has often been quantified using the thiobarbituric acid reactive substances (TBARSs) assay, which is widely used but lacks specificity because it captures a broader pool of reactive aldehyde-like products and is sensitive to pre-analytical handling, storage conditions, diet, and generalized inflammatory/metabolic states [15]. Accordingly, elevated TBARS-derived MDA should be interpreted as a broad signal of oxidative/lipid peroxidation-related burden rather than a highly specific readout of a single lipid peroxidation pathway. When available, more specific approaches such as HPLC-based MDA quantification or F2-isoprostanes may provide stronger analytical validity, although schizophrenia-specific data for these measures remain more limited [15].

Notably, some oxidative damage signals are detectable even in first-episode, medication-naïve patients [32], strengthening the interpretation that they reflect disease-related biological changes (rather than merely the effects of antipsychotic medications). However, peripheral oxidative damage markers remain highly susceptible to confounding factors such as smoking, diet, body mass index, metabolic syndrome, sleep disturbances, and chronic inflammatory states. Therefore, their most defensible clinical use at present is as risk-enrichment or subgroup-identification tools, rather than as direct surrogates of brain redox status. In other words, peripheral oxidative markers may help identify patients with a higher probability of systemic oxidative/inflammatory burden, but they do not by themselves establish central target engagement. This limitation is especially important when considering redox-targeted interventions intended to act on brain mechanisms.

#### 3.1.2. Antioxidant Buffering and Defense Systems

In the peripheral antioxidant defense system, changes in glutathione (GSH) are considered to have important explanatory significance for pathological mechanisms. Studies show that whole-blood GSH levels are significantly reduced in drug-naïve first-episode patients with schizophrenia [17], which is consistent with the hypothesis of diminished antioxidant buffering capacity. Because GSH not only participates in scavenging peroxides and maintaining thiol homeostasis of redox-sensitive proteins, but is also potentially coupled to glutamatergic/NMDAR function, a decline in GSH levels can be regarded as a key node linking peripheral biological markers with abnormalities in central nervous system function [11].

In contrast, findings on the activities of antioxidant enzymes such as superoxide dismutase (SOD), catalase (CAT), and glutathione peroxidase (GPx) show substantial heterogeneity, with reports of decreased activity as well as records of compensatory increases [20,21]. This inconsistent pattern is more likely to reflect dynamic dysregulation of the antioxidant defense system rather than a simple state of normal function or functional loss. In the early stage of the illness or in some individuals, the antioxidant enzyme system may be upregulated in the short term as a compensatory response to oxidative stress; whereas in the context of a chronic course, higher oxidative load, or comorbid inflammation/metabolic disturbances, the system may exhibit relative depletion or regulatory imbalance.

Beyond peripheral biochemistry, the glutathione hypothesis is also supported by genetic evidence. Polymorphisms and regulatory variation in genes involved in glutathione synthesis, especially GCLC and GCLM, have been reported in association with schizophrenia risk, altered GSH-related biology, or increased vulnerability under oxidative challenge in at least some cohorts [33,34]. Although the genetic evidence is not uniform enough to support deterministic conclusions, it is conceptually important because it links redox dysregulation to relatively trait-like biological liability rather than only to illness state or peripheral confounding. In the context of stratification, such findings suggest that glutathione-related genetic variation may help define biologically actionable subgroups when interpreted together with circulating biomarkers and clinical phenotype.

### 3.2. Central Evidence: MRS and CSF

Evidence from cerebrospinal fluid and in vivo magnetic resonance spectroscopy suggests that central glutathione-related abnormalities may occur in schizophrenia, but the literature is heterogeneous rather than settled. Some studies report lower CSF or medial prefrontal GSH, including in early or antipsychotic-naive samples [18], whereas others report no significant group difference or even higher regional GSH depending on field strength, acquisition method, brain region, illness stage, medication exposure, and analytic approach [19]. Therefore, brain GSH should be viewed as a mechanistically important but stage- and context-dependent measure, not as a uniformly reduced biomarker across schizophrenia.

### 3.3. Synthesis of Evidence

Taken together, these findings indicate that schizophrenia is characterized by both increased oxidative damage and dysregulated antioxidant defense. Among peripheral measures, lipid peroxidation signals (particularly MDA and 4-HNE) are among the most repeatedly reported abnormalities at the group level, though this conclusion is moderated by assay heterogeneity and specificity limitations—particularly for TBARS-based MDA measurements. The GSH axis may carry greater mechanistic relevance despite less uniform directionality across compartments and stages, while antioxidant enzyme activities display dynamic, stage-dependent patterns consistent with compensation-exhaustion transitions.

A key implication of this evidence profile is that peripheral biomarkers should not be interpreted as simple readouts of central redox pathology. Rather, they are better viewed as pragmatic but imperfect indicators of biological context that may enrich for subgroups in whom brain redox-related mechanisms are more likely to be relevant. Future translational work therefore needs to test, rather than assume, peripheral–central concordance by combining peripheral panels with more proximal measures such as MRS-GSH, CSF markers where feasible, or imaging markers related to white matter integrity and network dysfunction.

## 4. Multiscale Mechanistic Integration of Redox Dysregulation

Oxidative stress in schizophrenia should not be viewed merely as an isolated biochemical abnormality; rather, it may represent a self-sustaining pathological network node that interacts with other pathological processes across multiple scales. Within this network, redox imbalance is coupled with mitochondrial bioenergetic deficits and innate immune signaling, forming feedback loops whose downstream effects may exhibit selective vulnerability depending on cell type, ultimately mapping onto clinical phenotypes in the form of impaired excitation–inhibition balance, myelin and connectivity abnormalities, and reduced large-scale synchronization. The evidentiary status of each link differs: comparatively supported human observations (oxidative damage, GSH-related dysregulation, mitochondrial alterations, inflammatory activation, PV interneuron abnormalities, and oligodendrocyte/myelin involvement) are distinguished throughout this section from candidate amplification pathways (cGAS-STING/mtDAMP, nitrosative/peroxynitrite stress, GPx4-ferroptosis) that require direct validation in schizophrenia. The overall framework is summarized with evidence-tier annotations in Section 4.8.

### 4.1. Redox–Mitochondrial Coupling

Mitochondria serve as both a critical source of ROS and a key target of oxidative damage, thereby occupying a central position in the amplification and perpetuation of redox dysregulation. Electron leakage from the electron transport chain during oxidative phosphorylation can generate superoxide anions and their derivative ROS [35]. When mitochondrial efficiency declines or metabolic demands increase, ROS production can be significantly enhanced. Excessive ROS, in turn, can damage mitochondrial DNA, respiratory chain proteins, and membrane lipids, reduce ATP production, and disrupt membrane potential, forming a positive feedback loop of ROS-mitochondrial damage. Processes such as calcium overload, abnormalities in mitochondrial fusion–fission dynamics, and defective mitophagy can further compromise mitochondrial quality control and amplify ROS release [36].

Accumulating evidence from human and model studies supports the existence of alterations in schizophrenia consistent with mitochondrial dysfunction. Postmortem and functional studies have reported reduced activity or altered expression of respiratory chain components (particularly complexes I and IV) in disease-relevant brain regions [37], and metabolic phenotypes (e.g., lactate levels, tissue pH, glucose utilization) also suggest decreased bioenergetic efficiency [38,39,40]. Furthermore, increased oxidative damage and abnormalities in mitochondrial-related markers at the peripheral level provide additional support that this is not a phenomenon confined to a single compartment [41,42]. In mechanistic terms, the significance of the mitochondria–redox coupling lies in its persistence: chronic, low-grade but sustained metabolic-oxidative burden can accumulate during critical developmental periods, progressively reducing the resilience of specific cellular systems, thereby better aligning with the long-term trajectory disease characteristics of schizophrenia.

### 4.2. Redox–Immune Interactions

Mitochondrial damage not only leads to bioenergetic deficits but also activates innate immune pathways through the release of mitochondrial-derived damage-associated molecular patterns (mtDAMPs), such as mitochondrial DNA, cardiolipin, N-formyl peptides, and TFAM [43]. These signals can trigger inflammatory gene expression either intracellularly or extracellularly, thereby translating metabolic stress into immune responses. Among these, cytosolic mtDNA-induced inflammatory gene expression via the cGAS-STING axis represents a mechanistically highly plausible candidate pathway [44]. However, direct human evidence for this specific pathway in schizophrenia remains limited, and it should currently be regarded as a testable hypothesis rather than an established disease mechanism. Concurrently, mitochondrial-derived ROS can more broadly enhance inflammatory cascades such as NF-κB and promote cytokine production [45,46]. Conversely, inflammatory mediators such as TNF-α and IL-6 can impair mitochondrial respiratory efficiency, promote ROS generation, and disrupt antioxidant homeostasis [47]. Therefore, oxidative stress and inflammation are not parallel processes but constitute a redox-sensitive immune-metabolic feedback loop, the intensity and duration of which exhibit heterogeneity across different patients and disease stages, thereby providing a biological basis for phenotypic diversity.

Microglia may serve as critical effector and amplification nodes within this feedback loop. Under elevated oxidative burden or increased damage signals, microglial activation can release additional pro-inflammatory mediators, further elevating local oxidative stress [48]. Mechanisms such as the NLRP3 inflammasome provide candidate molecular mechanisms for the convergence of oxidative and inflammatory signals [49], but evidence for their involvement in schizophrenia similarly exhibits the characteristic of being mechanistically attractive but limited in terms of direct human data.

### 4.3. Nitrosative Stress as a Candidate Amplification Mechanism

The existing schizophrenia redox literature has predominantly focused on ROS and the GSH axis; however, at the biochemical level, reactive nitrogen species (RNS) may constitute a dimension that cross-couples with ROS and mutually amplifies their effects. NO and superoxide anion (O_2_^−^) can rapidly generate peroxynitrite anion (ONOO^−^), which can promote protein tyrosine nitration, lipid oxidation, and inhibition of mitochondrial respiratory chain function [50,51], thereby amplifying cellular stress in the context of metabolic abnormalities and inflammation. Since nitration and related RNS modifications can alter the structural and functional states of certain receptors, transporters, and key metabolic enzymes, the oxidation–nitration coupling may not only increase the damage burden but also modify the sensitivity of synaptic and immune signaling to stimuli.

It should be noted with caution: although nitration/nitrosative stress possesses clear biological plausibility in neuropsychiatric disorders, in schizophrenia, systematic and reproducible evidence regarding specific nitration markers, particularly those in the central compartment, remains insufficient [52]. Therefore, this review positions it as a candidate amplification pathway: its value lies in proposing testable predictions—that in patient subgroups with combined inflammatory-oxidative phenotypes, if stronger ONOO^−^-related nitration burden exists, it may be more closely associated with declined mitochondrial efficiency, cognitive/negative symptom outcomes, and may influence differential responses to antioxidant or anti-inflammatory strategies.

Taken together, these findings support the view that oxidative stress and innate immune dysregulation are functionally interconnected in schizophrenia. This framework may help explain the low-grade inflammatory profile repeatedly reported in the disorder, while also highlighting the need for caution when moving from general association to pathway-specific mechanistic claims.

### 4.4. Selective Cellular Vulnerability

Although oxidative stress may affect the brain broadly, its pathological impact is unlikely to be uniformly distributed across different neural cell types. A more explanatory model posits that chronic stress generated by oxidative–mitochondrial–immune coupling would first impact cell systems with high metabolic demands, limited antioxidant buffering capacity, or those rich in lipids/iron. In schizophrenia-related evidence, parvalbumin-positive (PV) interneurons and oligodendrocyte lineage cells are most frequently proposed as vulnerable targets, with their significance lying in providing intermediate-level mechanisms for how molecular-level abnormalities translate into circuit-level phenotypes. However, this should not be interpreted as exclusivity. Astrocytes, pyramidal neurons, and neuron-glia metabolic interactions are also highly relevant to redox homeostasis. A more balanced model is that schizophrenia-related oxidative stress operates within a multicellular network in which vulnerability depends not only on intrinsic cellular demand, but also on support functions, synaptic coupling, developmental timing, and circuit context.

#### 4.4.1. PV Interneurons

PV interneurons, as fast-spiking inhibitory neurons, exhibit continuous high-frequency electrical activity that results in extremely high energy demands and mitochondrial metabolic load, thereby demonstrating high dependence on strict regulation of redox homeostasis. In schizophrenia and related models, PV neuron-specific marker expression is reduced, cellular integrity is diminished, and this is accompanied by increased oxidative damage [12,53]. Given the central role of PV interneurons in maintaining cortical gamma oscillation synchrony and excitation–inhibition balance, their redox-related dysfunction provides a direct mechanistic pathway for cognitive deficits and abnormal network oscillations.

Furthermore, the antioxidant resilience of PV neurons is partially dependent on GSH precursors such as cysteine provided by astrocytes through the xCT system [54,55]. Therefore, disruption of astrocyte–interneuron metabolic coupling may further compromise the stability of PV cells under oxidative load, forming a cascade reaction from glial support abnormalities to inhibitory network dysfunction. In this context, astrocytes are particularly important because they help maintain extracellular glutamate balance, provide cysteine/cystine-related support for glutathione metabolism, and shape the redox resilience of nearby neurons. Likewise, pyramidal neurons should not be regarded as passive background elements: oxidative stress that affects excitatory neurons or their dendritic/synaptic function may also directly contribute to excitatory–inhibitory imbalance. Therefore, emphasizing PV interneurons should be understood more as highlighting a mechanistically sensitive node within a broader cellular ecosystem, rather than treating them as the sole cellular target of redox dysregulation.

#### 4.4.2. Oligodendrocyte Lineage Cells and Myelination

Oligodendrocyte precursor cells and mature oligodendrocytes may represent another selectively vulnerable population. Oligodendrocytes and their precursor cells are rich in lipid membranes, have relatively high iron metabolic demands, and possess relatively limited antioxidant buffering capacity, rendering them more susceptible to lipid peroxidation damage under elevated oxidative load or GSH deficiency [56]. Under conditions of glutathione depletion or excessive ROS exposure, oligodendrocyte precursor differentiation and myelin maturation may be disrupted. Structural imaging and postmortem studies in schizophrenia consistently report reduced white matter integrity and oligodendrocyte-related abnormalities [57,58,59]. Although oxidative stress is unlikely to account for all white matter alterations, it provides a critical mechanistic framework linking developmental windows (myelin maturation during adolescence to early adulthood) with metabolic-inflammatory stress.

### 4.5. Lipid Peroxidation–GPx4–Ferroptosis as a Candidate Amplification Mechanism

Lipid peroxidation represents one of the most characteristic downstream molecular events of oxidative stress, and its homeostasis is strictly regulated by GPx4. Utilizing GSH as a reducing agent, GPx4 specifically reduces hydroperoxides in membrane phospholipids, thereby blocking the propagation of lipid-free radical chain reactions [60,61]. When intracellular GSH is depleted or GPx4 activity is insufficient, lipid peroxidation may surpass the endogenous inhibitory threshold, subsequently triggering an iron-dependent cell death program characterized by oxidative damage to polyunsaturated fatty acid-containing phospholipids, known as ferroptosis [62,63]. In schizophrenia, this pathway holds particular mechanistic appeal: it not only aligns with the repeatedly reported elevations of lipid peroxidation markers in peripheral studies but also provides a more biochemically specific explanation for the selective vulnerability of the oligodendrocyte lineage and myelin system. This cell lineage is enriched in polyunsaturated fatty acid-containing membranes and exhibits high iron metabolic demands, rendering it particularly sensitive to lipid peroxidation imbalance. However, direct human evidence confirming the occurrence of ferroptosis in the brains of patients with schizophrenia remains limited at present. While more robust peripheral evidence suggests elevated lipid peroxidation, central nervous system validation is predominantly indirect. Therefore, this paper characterizes it as a set of testable candidate amplification mechanisms rather than an established disease pathway.

### 4.6. Dopamine Oxidation and Pharmacological Modulation of Redox State

Two schizophrenia specific sources of redox perturbation are easily overlooked when oxidative stress is treated generically. First, dopamine is itself pro-oxidant: its enzymatic and auto-oxidation generates dopamine-o-quinone, aminochrome and related quinones together with superoxide and hydrogen peroxide, and these species inhibit mitochondrial complex I, deplete GSH and impose oxidative load on catecholaminergic and neighbouring cells [64]. Dopaminergic hyperactivitymay therefore feed the very redox imbalance described here, providing a disease-specific rather than generic link between neurotransmission and oxidative burden. Second, antipsychotic drugs are not redox-neutral. First-generation agents (e.g., haloperidol) tend to lower antioxidant-enzyme activity and increase lipid peroxidation, whereas several second-generation agents (e.g., clozapine, olanzapine) display net antioxidant effects in experimental and some clinical settings; meta-analytic data confirm that antipsychotic exposure measurably shifts blood redox markers, with direction depending on drug class [65]. Consequently, antipsychotic treatment is a major confounding factor in peripheral and chronic-phase measurements; at the same time, medication itself constitutes an active and uncontrolled redox intervention, which must be taken into account in any trials of additional antioxidants.

### 4.7. From Cellular Injury to Circuit Dysfunction

The value of selective cellular vulnerability lies in establishing cross-scale mechanistic bridges. If redox imbalance preferentially impairs PV interneurons, inhibitory timing control would decline, excitation–inhibition balance would shift, and gamma oscillation synchrony would deteriorate, thereby more directly pointing to deficits in working memory, executive function, and information integration [66,67]. If oligodendrocyte lineage and myelin integrity are compromised, long-range conduction efficiency and cross-regional synchrony may be reduced, subsequently manifesting as connectivity abnormalities and distributed network dysfunction [68,69]. The redox-inflammatory amplification by microglia may alter synaptic homeostasis and pruning thresholds during critical developmental periods, further weakening network robustness.

Therefore, within this framework, oxidative stress does not explain schizophrenia phenotypes through diffuse, nonspecific damage, but rather transforms into circuit-level instability through selective disruption of critical “communication infrastructure”—inhibitory rhythms and myelinated conduction.

### 4.8. An Integrated Multi-Scale Model

**Figure 2:**
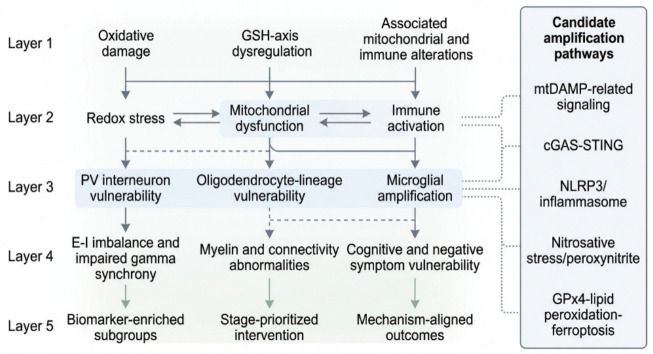
Evidence-weighted multiscale model of redox–mitochondria–immune dysregulation in schizophrenia. Peripheral oxidative damage, glutathione-axis dysregulation, and associated mitochondrial and immune alterations converge within an interacting redox–mitochondria–immune network. This network may disproportionately affect parvalbumin interneurons and oligodendrocyte-lineage cells, with microglia acting mainly as amplification nodes, thereby contributing to excitation–inhibition imbalance, impaired gamma synchrony, myelin/connectivity abnormalities, and vulnerability of cognitive and negative symptom domains. The figure also highlights translational implications, including biomarker-enriched subgrouping, stage-prioritized intervention, and mechanism-aligned outcomes. Candidate amplification pathways are shown separately to distinguish limited-direct-evidence mechanisms from relatively better-supported links in schizophrenia.

Current evidence most robustly supports oxidative damage, GSH-related dysregulation, and their association with mitochondrial and immune abnormalities in schizophrenia (Figure 2). These lines of evidence support a model in which redox dysregulation is functionally coupled to mitochondrial inefficiency and inflammatory activation and may contribute to circuit-level abnormalities through excitation–inhibition imbalance and myelin/connectivity disruption. At the same time, several pathway-specific extensions remain candidate amplification mechanisms rather than established schizophrenia pathways. These include mtDAMP/cGAS–STING signaling, nitrosative stress/peroxynitrite-related injury, and the GPx4–lipid peroxidation–ferroptosis axis. Their value in the present framework lies not in confirming their operation in schizophrenia, but in generating testable predictions regarding which biomarker-defined subgroups, cell types, and outcome domains may prove most informative in future longitudinal and translational studies.

Thus, the integrated model advanced here is intentionally evidence-weighted: it treats the redox–mitochondria–immune network as a useful organizational scaffold, while preserving a clear distinction between comparatively supported human observations and mechanistically plausible but still provisional links.

## 5. Stage Specificity and Clinical Heterogeneity

When redox abnormalities are situated within the “redox–mitochondria–immune” coupling network, a critical question emerges: is redox dysregulation a stable, unified characteristic throughout the disease process, or a stratified phenotype that dynamically varies across stages, symptom dimensions, and biological contexts? Existing evidence more strongly supports the latter. The abnormalities in oxidative damage and antioxidant defense are not constant between high-risk/prodromal phases, first-episode psychosis, and chronic schizophrenia. Rather, their association with clinical phenotypes is more likely to manifest as a pattern of being more relevant to certain outcome domains and more prominent in certain patients, rather than providing a uniform explanation across all symptom dimensions.

### 5.1. Stage-Specific Redox Dynamics

#### 5.1.1. High-Risk and Prodromal States

Elevated oxidative damage markers and early alterations in antioxidant defense have been reported in individuals at ultra-high risk (UHR) or during the prodromal phase [70]. These findings suggest that redox dysregulation may emerge prior to the onset of overt psychosis and may interact with genetic susceptibility and environmental stressors to collectively influence the risk of psychotic conversion.

However, compared with the chronic phase, the redox state during the prodromal phase is more likely to exhibit an unstable and partially compensated pattern: some studies indicate that the antioxidant system remains mobilizable or shows significant inter-individual variability, whereas others have observed measurable declines in glutathione (GSH)-related defenses [71]. Therefore, the critical focus of prodromal phase research is not simply to demonstrate the presence or absence of oxidative stress, but rather to identify specific redox phenotypes that have shifted toward decompensation in certain individuals, thereby providing a potential window for prediction and early intervention.

#### 5.1.2. First-Episode Psychosis

The first-episode psychosis (FEP) phase is frequently regarded as a period characterized by more pronounced redox imbalance: compared with controls, FEP patients more frequently exhibit increased oxidative damage and decreased antioxidant reserves [71,72], and multiple studies have linked these abnormalities to symptom severity or cognitive deficits [73]. Since these findings have also been observed in antipsychotic-naïve or minimally treated samples, they are unlikely to be entirely attributable to long-term medication exposure or chronic disease progression.

From the mechanistic framework outlined above, the significance of FEP lies in the fact that redox abnormalities during this phase may more readily co-occur with declined mitochondrial efficiency, immune activation, and cellular-type vulnerability, thereby being more likely to generate consequences detectable at the circuit level, such as alterations in synchrony, connectivity, or white matter metrics [74]. Therefore, FEP represents a more suitable critical phase for examining whether redox abnormalities possess mechanistic and clinical operability.

#### 5.1.3. Chronic Schizophrenia

In the chronic schizophrenia phase, redox abnormalities tend to become more entrenched, yet their interpretation is more complex. Peripheral and central indices during this phase may simultaneously reflect the persistent impact of primary disease mechanisms, cumulative stress from recurrent episodes, metabolic comorbidities and lifestyle burdens (such as smoking, diet, physical inactivity, sleep disturbances), as well as the metabolic effects of long-term antipsychotic medication exposure. Some studies suggest that the antioxidant enzyme system in the chronic phase tends to exhibit exhaustion or fluctuating imbalance rather than the compensatory upregulation that may occur in earlier stages [71]. Therefore, sustained oxidative burden may participate in maintaining cellular stress states and promoting long-term cognitive impairment [75].

### 5.2. Redox Patterns Across Symptom Domains

Redox abnormalities are not equally correlated with the positive, negative, and cognitive symptom dimensions of schizophrenia. Although statistical associations with multiple symptom domains are evident in the literature, from the perspective of cell types and circuit pathways, the most mechanistically consistent mapping leans more toward cognitive impairment and negative symptoms. Impaired PV interneuron function is more directly associated with gamma synchrony, information integration, and working memory/executive function; impairment of the oligodendrocyte lineage and myelin integrity more directly affects long-range communication efficiency, connectivity, and network stability. These processes more naturally point to persistent deficits in functional outcomes rather than the intensity of short-term fluctuating psychotic experiences [76].

### 5.3. Clinical Subgroups and Biological Stratification

The substantial heterogeneity of schizophrenia itself suggests that redox abnormalities are more likely to carry greater mechanistic weight in specific subgroups. Based on existing evidence and mechanistic plausibility, at least three categories of “working stratification hypotheses” may be proposed (emphasizing: these are not yet strict subtype definitions): the inflammation–oxidation coupling phenotype, wherein individuals simultaneously exhibiting low-grade inflammation and elevated oxidative damage may correspond to a stronger immune-metabolic feedback loop; the bioenergetic/mitochondrial phenotype, wherein those characterized by prominent mitochondrial efficiency decline and metabolic abnormalities may have redox abnormalities more closely coupled with energy insufficiency; and the cognitive/connectivity vulnerability phenotype, wherein individuals with prominent cognitive deficits, persistent negative symptoms, or poor functional outcomes may be enriched for redox imbalance signals related to the PV-myelin-network synchrony cascade.

### 5.4. Summary

The framework adopted in this review emphasizes that redox abnormalities in schizophrenia are more likely to represent a dynamic, stratified phenotype that evolves with disease stages: the prodromal phase may exhibit unstable or partially compensated signals, first-episode psychosis (FEP) may represent a critical window that is more detectable and amenable to intervention, whereas the chronic phase, due to cumulative burden and multiple confounders, renders causal inference more challenging. Clinically, redox-related pathology is more likely to map to cognitive and negative symptom dimensions and, through the selective vulnerability of PV interneurons and oligodendrocyte lineage, to circuit synchrony and connectivity abnormalities. These conclusions directly inform subsequent treatment chapters: the evaluation of antioxidant or redox modulation strategies should employ stage-sensitive enrollment criteria, actionable biomarker stratification, and mechanism-relevant outcome measures to avoid diluting true effects in unstratified samples.

## 6. Therapeutic Translation: From Nonspecific Antioxidant Supplementation to Stratified, Stage-Sensitive, and Mechanism-Informed Interventions

Building on the preceding sections, this section no longer addresses the question of whether antioxidant treatment is effective in general, but instead focuses on three questions with greater translational significance: which interventions demonstrate relatively more credible clinical signals? Why do existing trials often yield mixed or even negative results? How can future studies improve the testability and reproducibility of redox-targeted strategies through biomarker stratification, stage-sensitive enrollment, and mechanism-relevant outcomes?

### 6.1. Current Evidence for Redox-Targeted Interventions

Representative clinical studies on these interventions are summarized in Table 2.

#### 6.1.1. NAC

N-acetylcysteine (NAC) is currently the most extensively studied compound in redox interventions for schizophrenia, with its primary mechanism involving the provision of cysteine precursors to facilitate GSH synthesis, while potentially influencing inflammatory and glutamatergic homeostasis. Early randomized controlled trials and systematic reviews indicated that longer treatment courses (typically ≥24 weeks) of NAC may produce improvement signals in negative symptoms and certain cognitive domains, such as working memory and information processing speed [77]. However, longer-term or larger-sample studies have not consistently replicated these findings. For instance, a 52-week multicenter trial and recent network meta-analyses failed to observe significant and consistent symptomatic benefits [78,79]. These mixed findings do not eliminate the mechanistic rationale for NAC, but they also caution against overstating its clinical promise. A reasonable interpretation is that redox-targeted treatment effects in schizophrenia may be modest, subgroup-dependent, or limited to particular outcome domains, rather than broadly effective across unselected populations. Better stratification, target-engagement assessment, and mechanism-aligned outcomes may improve signal detection, but future negative trials under such conditions would weigh substantially against strong therapeutic claims.

#### 6.1.2. SFN

Sulforaphane (SFN) upregulates endogenous antioxidant and cytoprotective genes through activation of the Nrf2 pathway, thereby theoretically representing a more systematic approach to redox modulation compared to “simple antioxidant supplementation.” Existing trials and meta-analyses have suggested potential improvement signals for negative symptoms [80,81]. However, the durability and clinical consistency of these effects remain uncertain, and current evidence is insufficient to establish a clear temporal-window pattern [82]. At most, the available data are compatible with the hypothesis that SFN may be more relevant in earlier or biologically more responsive subgroups, which should be tested prospectively rather than inferred conclusively from current meta-analytic evidence.

#### 6.1.3. Omega-3, Vitamins, and Other Strategies

Omega-3 polyunsaturated fatty acids have demonstrated symptomatic improvement accompanied by reductions in oxidative stress markers in certain trials, particularly exhibiting greater promise in early-stage or first-episode samples [83]. However, these studies are characterized by substantial heterogeneity, with considerable variations in dosage, EPA/DHA ratios, baseline dietary backgrounds, and outcome selection, rendering it challenging to synthesize their effects into unified conclusions. The results from direct antioxidant strategies such as vitamins E and C further underscore the issue of stratification deficiency: under certain baseline lipid profiles, vitamin E supplementation alone may even be associated with worsening of sustained attention deficits, whereas partial amelioration occurs only when combined with fish oil [84,85]. At most, the available data are compatible with the hypothesis that SFN may be more relevant in earlier or biologically more responsive subgroups, which should be tested prospectively rather than inferred conclusively from current meta-analytic evidence. Regarding mitochondria-targeted strategies such as CoQ10, despite having a clear theoretical foundation, there is currently insufficient high-quality, reproducible randomized controlled trial evidence to support their clinical application value.

### 6.2. Why Have Clinical Results Been Inconsistent?

Multiple factors likely contribute to the mixed outcomes of existing trials, including biological heterogeneity, stage mismatch, limited target engagement assessment, and outcome measures that are insufficiently proximal to the proposed mechanisms. However, these design considerations should not be invoked to reflexively dismiss null findings. An equally tenable interpretation is that redox-targeted interventions, particularly when deployed as adjunctive or monotherapeutic strategies in schizophrenia, may yield only modest average effect sizes or may be clinically meaningful exclusively within specific subpopulations. Accordingly, negative trials should be regarded as evidence constraining the overgeneralization of therapeutic claims, even though they do not, in themselves, invalidate all mechanistic roles attributed to redox dysregulation.

Secondly, stage mismatch represents another critical factor. If redox interventions primarily act on early stress amplification, imbalance of cellular defense, and decline in circuit plasticity, then their most rational window of intervention should be during the ultra-high-risk/first-episode psychosis stage or at least during earlier phases of the illness. If intervention occurs during the chronic stage, against a background of long-term medication exposure and accumulated metabolic comorbidities, when some downstream consequences may have already transformed into difficult-to-reverse structural and network abnormalities, clinical improvement is naturally constrained. Therefore, prioritizing early stages should not merely be an empirical judgment but should serve as a research design principle aligned with the mechanistic model.

Thirdly, the mismatch between outcome selection and mechanistic chains further dilutes true effects. Redox imbalance is more likely to affect cognitive and negative dimensions through PV interneurons, oligodendrocyte lineage, and connectivity/synchronicity abnormalities. If total psychiatric symptom scores are used as the sole primary endpoint, this may inherently underestimate therapeutic efficacy. Outcomes more aligned with the mechanistic framework should prioritize cognitive domains (working memory, executive function, processing speed), functional outcomes, and mechanism-relevant imaging endpoints (white matter integrity, myelin-sensitive imaging, functional connectivity, MRS-GSH). This does not imply that positive symptoms are irrelevant, but rather that they are not the most prioritized or sensitive readouts.

### 6.3. Toward Biomarker-Guided and Stage-Specific Intervention

Based on the preceding evidence, a more promising strategy is not to employ antioxidants as a uniform adjunctive treatment, but to identify biologically actionable redox-related phenotypes. Importantly, such phenotypes should not be defined by assuming that peripheral markers directly mirror brain redox status. Instead, peripheral markers are better conceptualized as screening and enrichment tools that may increase the likelihood of recruiting patients with relevant oxidative-inflammatory burden, while central or mechanism-proximal measures are needed whenever feasible to assess target engagement.

In practical research, stratification may therefore proceed in two stages. A first-stage, pragmatic screen could enrich for candidate subgroups using accessible peripheral indicators, including lipid peroxidation markers, antioxidant buffering indices, and inflammatory markers. Among peripheral candidates with the strongest replication record in schizophrenia, the most practical markers for first-stage screening include blood GSH and GSH/GSSG ratio as indices of antioxidant buffering capacity; malondialdehyde (MDA), 4-hydroxynonenal (4-HNE), or F2-isoprostanes as lipid peroxidation markers; and high-sensitivity C-reactive protein (hs-CRP), interleukin-6 (IL-6), or tumor necrosis factor-alpha (TNF-α) as inflammatory indicators. On this basis, several provisional subgroup profiles can be envisioned for prospective testing. A high-oxidative-load/low-antioxidant-buffering profile is operationally defined by elevated lipid peroxidation markers together with reduced GSH/GSSG ratio. An inflammation–oxidation coupling profile is defined by concurrent elevation of oxidative damage markers and one or more inflammatory indicators. A bioenergetic-impairment profile is defined by oxidative markers combined with reduced mitochondrial function indices such as elevated blood lactate or reduced respiratory chain complex I activity. These profiles are intended as operational starting points for trial enrichment, not as definitive taxonomies; their predictive validity remains to be established through prospective studies that combine peripheral screening with mechanism-proximal confirmation measures.

### 6.4. Combination and Systems-Based Strategies

Given that redox abnormalities are embedded within a broader pathological network involving mitochondrial dysfunction, innate immune activation, myelin vulnerability, and network instability, the effects of single antioxidant strategies are often limited and context-dependent. A direction more aligned with systems biology logic is to regard redox modulation as one node within combination therapy rather than as an independent solution. For instance, future trials with greater value may consider the following combined approaches: redox modulation combined with mitochondrial support to target energy deficiency and ROS positive feedback loops; redox modulation combined with immunomodulation to address inflammation–oxidation coupling phenotypes; redox modulation combined with interventions promoting white matter plasticity to target oligodendroglial lineage vulnerability and connectivity abnormalities; and redox modulation combined with cognitive training interventions to enhance the utilization of plasticity at both network and behavioral levels while improving biological background noise. The core logic of this combinatorial approach lies in the fact that if redox dysregulation primarily serves as an amplifier rather than the sole driver, then merely reducing oxidative load alone may be insufficient to produce significant clinical effects, whereas simultaneous intervention at other nodes is more likely to reveal mechanistic and functional benefits.

### 6.5. Cell-Type-Informed Antioxidant Strategies

A particularly promising direction for therapeutic translation is to align antioxidant intervention with the selective cellular vulnerability observed in schizophrenia. If redox dysregulation does not affect all neural elements equally, then treatment strategies may also need to move beyond nonspecific antioxidant supplementation toward protection of the cell populations and biological processes that are most susceptible to oxidative injury. In this framework, antioxidant therapy is best understood not as a uniform disease-modifying strategy, but as a means of preserving vulnerable neural systems under specific biological and clinical conditions.

#### 6.5.1. Protecting Parvalbumin Interneurons

PV interneurons represent high-metabolism, redox-sensitive vulnerable nodes, whereby enhancing GSH buffering, strengthening endogenous antioxidant responses, or attenuating oxidative-inflammatory amplification may theoretically help maintain inhibitory rhythms and gamma synchrony. Preclinical studies have indicated that in GSH-deficient models, supplementation with GSH precursors or activation of Nrf2 can partially restore PV expression and gamma oscillations [12]. PV dysfunction is not caused solely by single oxidative damage but also involves pyramidal inputs, glial support, and network states [86]. Therefore, redox interventions are more likely to manifest as reducing the risk of further deterioration or enhancing the window of network plasticity, rather than independently restoring normal gamma oscillations.

#### 6.5.2. Protecting Oligodendrocyte Lineage Cells and Myelin Integrity

The oligodendrocyte lineage and myelin system, characterized by their lipid-rich composition, iron association, and relatively weak antioxidant buffering capacity, may represent important vulnerable targets under conditions of lipid peroxidation. Consequently, the therapeutic value of redox interventions may manifest not only in symptomatic improvement but also in preserving white matter integrity and maintaining long-range communication efficiency. This perspective aligns with findings from early psychosis studies demonstrating that NAC improves fornix white matter integrity, with effects correlating with prefrontal GSH indices [87]. Omega-3 and SFN/Nrf2 strategies warrant continued evaluation in this dimension due to their relevance to membrane lipid homeostasis and endogenous antioxidant regulation [88,89]. Future relevant trials should increasingly employ DTI, myelin-sensitive imaging, and connectivity measures as mechanistic endpoints, rather than relying solely on symptom rating scales.

#### 6.5.3. Modulating Microglial Redox–Inflammatory Amplification

Regarding microglia, the objective is to attenuate redox-inflammation amplification rather than simply suppress inflammation. Within this framework, microglia are not merely passive victims but rather active amplification nodes in the redox–immune coupling. Therefore, reducing mitochondrial ROS, limiting aberrant inflammasome activation, or attenuating mtDNA-driven innate immune amplification may theoretically provide indirect protection to more vulnerable neuronal and oligodendrocyte systems. However, this direction currently remains primarily a prospective translational hypothesis. Strategies such as NOX2 inhibition, NLRP3 modulation, and mitochondria-targeted antioxidants still have limited direct clinical evidence in schizophrenia [90,91,92,93]. Microglia-directed redox interventions are more appropriately positioned as key directions for future mechanistic advancement rather than currently validated therapeutic pathways.

Overall, existing clinical evidence suggests that antioxidant or redox modulation interventions in schizophrenia are not devoid of signals, but rather that signals are obscured by heterogeneity and design limitations. NAC and SFN provide the most representative proof of concept, yet the true value of their effects may not lie in demonstrating efficacy for all patients, but rather in advancing the field from non-specific supplementation toward the following pathways: biomarker-guided sample selection; intervention windows prioritizing first-episode stages; evaluation systems prioritizing cognitive, functional, and mechanistic endpoints; and systematic treatment models combining mitochondrial, immune, and myelin/network plasticity approaches. In other words, the key question is not whether antioxidants are uniformly useful, but under what biological conditions, at what disease stage, and for which outcomes redox-targeted strategies may have measurable value. It also remains possible that, even under improved designs, their effect sizes will prove limited, so an outcome that future stratified trials must be able to establish clearly.

## 7. Future Directions

The future of this field lies not in repeatedly demonstrating the presence of oxidative stress, but in determining its mechanistic and therapeutic significance in specific patient populations, at particular disease stages, and within specific cellular and circuit contexts. Accordingly, this section outlines research priorities organized around three themes: stratification, translational design, and cell-type-informed intervention.

### 7.1. Biomarker-Guided, Stage-Sensitive Stratification

Building on the stage-sensitive and biomarker-guided principles discussed in Section 5 and Section 6, future research should move from cross-sectional comparisons toward longitudinal designs that track co-evolutionary trajectories of oxidative damage, GSH status, inflammatory markers, and clinical outcomes across illness stages. Rather than seeking a single optimal marker, the priority is to validate combinatorial panels—peripheral lipid peroxidation, GSH-related indices, inflammatory profiles, and, where feasible, MRS-GSH, white matter integrity, and functional connectivity—that define actionable subgroups characterized by high oxidative load/low antioxidant buffering, inflammation-oxidation coupling, or bioenergetic impairment. Only such stratification frameworks can address which patients’ cognitive impairment, negative symptoms, or functional trajectories reflect genuine redox-mechanistic associations rather than epiphenomena of chronicity or comorbidity.

Another underdeveloped but important direction is the study of sex differences. Redox regulation, immune tone, mitochondrial function, and antipsychotic-related metabolic effects all show sex-dependent variation [94], and schizophrenia itself exhibits sex differences in age at onset, symptom profile, and course. Future stratification studies should therefore report sex-disaggregated biomarker patterns and test whether redox-targeted strategies differ in relevance or response across male and female patients.

### 7.2. Translating Stratification into Precise Redox-Targeted Therapy

To translate stratification into practice, future trials should adopt screen-and-confirm designs: peripheral biomarkers provide low-cost prescreening, while enrollment is refined using at least one mechanism-proximal readout (MRS-GSH, white matter integrity, or functional connectivity) when resources permit. Such designs directly test peripheral–central concordance rather than presuming it, and help determine whether peripheral oxidative signatures truly enrich for brain target engagement. Trials should prioritize prodromal and first-episode windows and employ mechanism-aligned outcomes—cognitive function, negative symptoms, functional measures, and imaging endpoints—alongside pre-post biomarker measurement to verify target engagement. Only through such designs can the field distinguish true treatment inefficacy from failures of patient selection or target engagement.

### 7.3. From Broad Antioxidant Supplementation to Cell-Type-Informed Interventions

As discussed in Section 6.5, the cell-type-informed framework maps onto three intervention targets: PV interneurons, oligodendrocyte/myelin integrity, and microglial redox-inflammatory amplification. The priority for future research is to translate this framework into testable combinatorial protocols and to incorporate the corresponding circuit-level readouts into prospective trial designs.

## 8. Conclusions

As outlined in Section 4.8, redox abnormalities in schizophrenia are best understood within an evidence-weighted framework: oxidative damage, glutathione-related dysregulation, and their coupling with mitochondrial and immune dysfunction are comparatively well-supported, while pathway-specific extensions, including cGAS-STING/mtDAMP signaling, nitrosative/peroxynitrite stress, and GPx4-ferroptosis remain candidate mechanisms that require direct validation in human schizophrenia.

Based on this framework, the focus of redox research should shift from describing average differences toward identifying subgroups with mechanistic and clinical significance. The more important question for future research is not whether oxidative stress exists in schizophrenia, but rather which patients exhibit actionable redox abnormalities at which disease stages, and which cells, circuits, and clinical outcomes are most closely associated with these abnormalities. This also implies that redox-targeted therapies should no longer remain at the level of non-specific antioxidant supplementation, but should rely more heavily on biomarker-guided stratification strategies, stage-sensitive intervention designs, and mechanism-informed treatments targeting vulnerable cellular systems.

In summary, the true value of redox biology in schizophrenia does not lie in adding another broad pathological label to the disease, but in providing an integrative framework that spans molecular, cellular, circuit, and clinical phenotypes for understanding its heterogeneity. If future research can combine stratification studies with cell-type-informed therapeutic development, redox abnormalities hold promise for transforming from descriptive correlation phenomena into practical pathways supporting precision stratification and personalized intervention in schizophrenia.

## Figures and Tables

**Table 1:** Key oxidative stress biomarkers in schizophrenia.

Biomarker	Category	Direction of Change	Tissue/Source	Studies/Consistency	Key Interpretation	Tier (E/M/X)
MDA	Lipid peroxidation	↑ Elevated	Plasma, erythrocytes	Multiple meta-analyses; >10 independent studies	Consistent marker of membrane oxidative damage; Multiple meta-analyses; ↑ (assay-dependent, largely TBARS)	E
4-HNE	Lipid peroxidation	↑ Elevated	Plasma, erythrocytes	Several studies; consistent direction	Aldehyde product; biomarker of lipid peroxidation	E/M
AOPP	Protein oxidation	↑ in some studies	Plasma	Few studies; less consistent	Indicates systemic oxidative burden; Few studies; less consistent	M
GSH (total)	Non-enzymatic antioxidant	↓ peripheral; brain	Whole blood, PFC (MRS), CSF	Multiple studies; peripheral fairly consistent, central mixed	Impaired intracellular antioxidant reserve; Peripheral ↓ fairly consistent; central GSH mixed	E/M
SOD	Enzymatic antioxidant	Variable (↑/↓/↔)	Erythrocytes, plasma	Many studies; highly variable across illness stages	Dynamically dysregulated across illness stages	M
CAT	Enzymatic antioxidant	Variable	Erythrocytes, plasma	Several studies; inconsistent	Inconsistent findings; possible compensation	M
GPx	Enzymatic antioxidant	Variable	Erythrocytes	Several studies; heterogeneous	Heterogeneous; tissue- and stage-dependent	M
Isoprostanes	Lipid peroxidation	↑ (general LPO gold standard)	Plasma, CSF, urine	Few SCZ-specific studies; inconsistent	Gold standard for in vivo oxidative stress; SCZ-specific data are sparse and not uniformly positive	M/X

Tiers: E = replicated human/meta-analytic; M = animal/model-supported or mixed human; X = extrapolated. Arrows indicate direction of change relative to controls: ↑, elevated; ↓, reduced; ↔, unchanged; ↑/↓/↔, variable or inconsistent. Representative references: MDA/4-HNE [13,14,15]; AOPP [16]; GSH [17,18,19]; SOD/CAT/GPx [20,21]; isoprostanes [15].

**Table 2:** Representative redox-targeted interventions in schizophrenia.

Agent	Duration (Typical)	Evidence Tier	Result (Concise)
N-acetylcysteine (NAC)	24–52 weeks	E	Mixed; benefits in some early/non-resistant subgroups
Sulforaphane (SFN)	24 weeks	M	Possible early signals for negative symptoms; durability and consistency uncertain
n-3 PUFAs (Omega-3)	~26 weeks	M	Modest benefit in early/first-episode samples
Vitamins E + C	16–26 weeks	M	Inconsistent; possible harm in specific subgroups
CoQ10/mitochondria-targeted agents	—	X	Theoretical promise; RCT evidence lacking
Targeted redox/inflammasome strategies (NOX2, NLRP3, etc.)	—	X	Preclinical/early phase; clinical data limited

Tiers: E = replicated human/meta-analytic; M = animal/model-supported or mixed human; X = extrapolated.

## Data Availability

No new data were created or analyzed in this study. Data sharing is not applicable to this article.

## References

[1] Borelli C.M., Solari H. (2019). Schizophrenia. JAMA.

[2] Muszynski J., Bienert A., Elsorady R.W., Rybakowski F. (2025). New pharmacological approaches in the treatment of schizophrenia. Pharmacol. Rep..

[3] Murray A.J., Rogers J.C., Katshu M., Liddle P.F., Upthegrove R. (2021). Oxidative Stress and the Pathophysiology and Symptom Profile of Schizophrenia Spectrum Disorders. Front. Psychiatry.

[4] Wiedlocha M., Zborowska N., Marcinowicz P., Debowska W., Debowska M., Zalewska A., Maciejczyk M., Waszkiewicz N., Szulc A. (2023). Oxidative Stress Biomarkers among Schizophrenia Inpatients. Brain Sci..

[5] Rajasekaran A., Venkatasubramanian G., Berk M., Debnath M. (2015). Mitochondrial dysfunction in schizophrenia: Pathways, mechanisms and implications. Neurosci. Biobehav. Rev..

[6] Upthegrove R., Khandaker G.M. (2020). Cytokines, Oxidative Stress and Cellular Markers of Inflammation in Schizophrenia. Curr. Top. Behav. Neurosci..

[7] Cuenod M., Steullet P., Cabungcal J.H., Dwir D., Khadimallah I., Klauser P., Conus P., Do K.Q. (2022). Caught in vicious circles: A perspective on dynamic feed-forward loops driving oxidative stress in schizophrenia. Mol. Psychiatry.

[8] Maas D.A., Valles A., Martens G.J.M. (2017). Oxidative stress, prefrontal cortex hypomyelination and cognitive symptoms in schizophrenia. Transl. Psychiatry.

[9] Cecerska-Heryc E., Polikowska A., Serwin N., Michalczyk A., Stodolak P., Goszka M., Zon M., Budkowska M., Tyburski E., Podwalski P. (2024). The importance of oxidative biomarkers in diagnosis, treatment, and monitoring schizophrenia patients. Schizophr. Res..

[10] Goh X.X., Tang P.Y., Tee S.F. (2022). Blood-based oxidation markers in medicated and unmedicated schizophrenia patients: A meta-analysis. Asian J. Psychiatr..

[11] Steullet P., Cabungcal J.H., Monin A., Dwir D., O’Donnell P., Cuenod M., Do K.Q. (2016). Redox dysregulation, neuroinflammation, and NMDA receptor hypofunction: A “central hub” in schizophrenia pathophysiology?. Schizophr. Res..

[12] Steullet P., Cabungcal J.H., Coyle J., Didriksen M., Gill K., Grace A.A., Hensch T.K., LaMantia A.S., Lindemann L., Maynard T.M. (2017). Oxidative stress-driven parvalbumin interneuron impairment as a common mechanism in models of schizophrenia. Mol. Psychiatry.

[13] Flatow J., Buckley P., Miller B.J. (2013). Meta-analysis of oxidative stress in schizophrenia. Biol. Psychiatry.

[14] Saidane L., Medjati N.D., Harek Y., Rouabhi H., Guermouche B., Benosman C., Sahi M.D. (2025). Peripheral Oxidative Stress Markers in Schizophrenia: Insights from Erythrocyte Glutathione Peroxidase, Plasma Malondialdehyde, and Trace Element Ratios in Algerian Patients. Biol. Trace Elem. Res..

[15] Tsikas D. (2017). Assessment of lipid peroxidation by measuring malondialdehyde (MDA) and relatives in biological samples: Analytical and biological challenges. Anal. Biochem..

[16] Maes M., Sirivichayakul S., Matsumoto A.K., Michelin A.P., de Oliveira Semeao L., de Lima Pedrao J.V., Moreira E.G., Barbosa D.S., Carvalho A.F., Solmi M. (2020). Lowered Antioxidant Defenses and Increased Oxidative Toxicity Are Hallmarks of Deficit Schizophrenia: A Nomothetic Network Psychiatry Approach. Mol. Neurobiol..

[17] Raffa M., Atig F., Mhalla A., Kerkeni A., Mechri A. (2011). Decreased glutathione levels and impaired antioxidant enzyme activities in drug-naive first-episode schizophrenic patients. BMC Psychiatry.

[18] Murray A.J., Humpston C.S., Wilson M., Rogers J.C., Katshu M.Z.U.H., Liddle P.F., Upthegrove R. (2024). Measurement of brain glutathione with magnetic Resonance spectroscopy in Schizophrenia-Spectrum disorders—A systematic review and Meta-Analysis. Brain Behav. Immun..

[19] Kumar J., Liddle E.B., Fernandes C.C., Palaniyappan L., Hall E.L., Robson S.E., Simmonite M., Fiesal J., Katshu M.Z., Qureshi A. (2020). Glutathione and glutamate in schizophrenia: A 7T MRS study. Mol. Psychiatry.

[20] Buosi P., Borghi F.A., Lopes A.M., Facincani I.D.S., Fernandes-Ferreira R., Oliveira-Brancati C.I.F., do Carmo T.S., Souza D.R.S., da Silva D.G.H., de Almeida E.A. (2021). Oxidative stress biomarkers in treatment-responsive and treatment-resistant schizophrenia patients. Trends Psychiatry Psychother..

[21] Djordjevic V.V., Kostic J., Krivokapic Z., Krtinic D., Rankovic M., Petkovic M., Cosic V. (2022). Decreased Activity of Erythrocyte Catalase and Glutathione Peroxidase in Patients with Schizophrenia. Medicina.

[22] Beckhauser T.F., Francis-Oliveira J., De Pasquale R. (2016). Reactive Oxygen Species: Physiological and Physiopathological Effects on Synaptic Plasticity. J. Exp. Neurosci..

[23] Clemente-Suarez V.J., Martin-Rodriguez A., Curiel-Regueros A., Rubio-Zarapuz A., Tornero-Aguilera J.F. (2025). Neuro-Nutrition and Exercise Synergy: Exploring the Bioengineering of Cognitive Enhancement and Mental Health Optimization. Bioengineering.

[24] Lee K.H., Cha M., Lee B.H. (2020). Neuroprotective Effect of Antioxidants in the Brain. Int. J. Mol. Sci..

[25] Singh A., Kukreti R., Saso L., Kukreti S. (2019). Oxidative Stress: A Key Modulator in Neurodegenerative Diseases. Molecules.

[26] Collin F. (2019). Chemical Basis of Reactive Oxygen Species Reactivity and Involvement in Neurodegenerative Diseases. Int. J. Mol. Sci..

[27] Wu W.L., Gong X.X., Qin Z.H., Wang Y. (2025). Molecular mechanisms of excitotoxicity and their relevance to the pathogenesis of neurodegenerative diseases-an update. Acta Pharmacol. Sin..

[28] Trofin D.M., Sardaru D.P., Trofin D., Onu I., Tutu A., Onu A., Onita C., Galaction A.I., Matei D.V. (2025). Oxidative Stress in Brain Function. Antioxidants.

[29] Radi R. (2018). Oxygen radicals, nitric oxide, and peroxynitrite: Redox pathways in molecular medicine. Proc. Natl. Acad. Sci. USA.

[30] Yakovlev V.A., Mikkelsen R.B. (2010). Protein tyrosine nitration in cellular signal transduction pathways. J. Recept. Signal Transduct. Res..

[31] Chen Z., Muscoli C., Doyle T., Bryant L., Cuzzocrea S., Mollace V., Mastroianni R., Masini E., Salvemini D. (2010). NMDA-receptor activation and nitroxidative regulation of the glutamatergic pathway during nociceptive processing. Pain.

[32] Dudzinska E., Szymona K., Bogucki J., Koch W., Cholewinska E., Sitarz R., Ognik K. (2022). Increased Markers of Oxidative Stress and Positive Correlation Low-Grade Inflammation with Positive Symptoms in the First Episode of Schizophrenia in Drug-Naive Patients. J. Clin. Med..

[33] Gysin R., Kraftsik R., Sandell J., Bovet P., Chappuis C., Conus P., Deppen P., Preisig M., Ruiz V., Steullet P. (2007). Impaired glutathione synthesis in schizophrenia: Convergent genetic and functional evidence. Proc. Natl. Acad. Sci. USA.

[34] Xin L., Mekle R., Fournier M., Baumann P.S., Ferrari C., Alameda L., Jenni R., Lu H., Schaller B., Cuenod M. (2016). Genetic Polymorphism Associated Prefrontal Glutathione and Its Coupling With Brain Glutamate and Peripheral Redox Status in Early Psychosis. Schizophr. Bull..

[35] Murphy M.P. (2009). How mitochondria produce reactive oxygen species. Biochem. J..

[36] Chu H., Cui C., Su X., Zhang H., Ma J., Zhu H., Bai L., Li R. (2024). Research progress in mitochondrial quality control in schizophrenia. Zhong Nan Da Xue Xue Bao Yi Xue Ban.

[37] Moren C., Olivares-Berjaga D., Martinez-Pinteno A., Bioque M., Rodriguez N., Gasso P., Martorell L., Parellada E. (2025). Mitochondrial Oxidative Phosphorylation System Dysfunction in Schizophrenia. Int. J. Mol. Sci..

[38] Liu S., Zhang L., Fan X., Wang G., Liu Q., Yang Y., Shao M., Song M., Li W., Lv L. (2024). Lactate levels in the brain and blood of schizophrenia patients: A systematic review and meta-analysis. Schizophr. Res..

[39] Caddye E., Pineau J., Reyniers J., Ronen I., Colasanti A. (2023). Lactate: A Theranostic Biomarker for Metabolic Psychiatry?. Antioxidants.

[40] Valiente-Palleja A., Torrell H., Alonso Y., Vilella E., Muntane G., Martorell L. (2020). Increased blood lactate levels during exercise and mitochondrial DNA alterations converge on mitochondrial dysfunction in schizophrenia. Schizophr. Res..

[41] Fizikova I., Dragasek J., Racay P. (2023). Mitochondrial Dysfunction, Altered Mitochondrial Oxygen, and Energy Metabolism Associated with the Pathogenesis of Schizophrenia. Int. J. Mol. Sci..

[42] Kumar P., Efstathopoulos P., Millischer V., Olsson E., Wei Y.B., Brustle O., Schalling M., Villaescusa J.C., Ösby U., Lavebratt C. (2018). Mitochondrial DNA copy number is associated with psychosis severity and anti-psychotic treatment. Sci. Rep..

[43] Zhang Q., Raoof M., Chen Y., Sumi Y., Sursal T., Junger W., Brohi K., Itagaki K., Hauser C.J. (2010). Circulating mitochondrial DAMPs cause inflammatory responses to injury. Nature.

[44] Caielli S., Athale S., Domic B., Murat E., Chandra M., Banchereau R., Baisch J., Phelps K., Clayton S., Gong M. (2016). Oxidized mitochondrial nucleoids released by neutrophils drive type I interferon production in human lupus. J. Exp. Med..

[45] Rius-Perez S., Torres-Cuevas I., Millan I., Ortega A.L., Perez S. (2020). PGC-1alpha, Inflammation, and Oxidative Stress: An Integrative View in Metabolism. Oxidative Med. Cell. Longev..

[46] Xu J., Wakai M., Xiong K., Yang Y., Prabakaran A., Wu S., Ahrens D., Molina-Portela M.D.P., Ni M., Bai Y. (2025). The pro-inflammatory cytokine IL6 suppresses mitochondrial function via the gp130-JAK1/STAT1/3-HIF1alpha/ERRalpha axis. Cell Rep..

[47] Morgan M.J., Liu Z.G. (2011). Crosstalk of reactive oxygen species and NF-kappaB signaling. Cell Res..

[48] Dwir D., Khadimallah I., Xin L., Rahman M., Du F., Ongur D., Do K.Q. (2023). Redox and Immune Signaling in Schizophrenia: New Therapeutic Potential. Int. J. Neuropsychopharmacol..

[49] Gober R., Dallmeier J., Davis D., Brzostowicki D., de Rivero Vaccari J.P., Cyr B., Barreda A., Sun X., Gultekin S.H., Garamszegi S. (2024). Increased inflammasome protein expression identified in microglia from postmortem brains with schizophrenia. J. Neuropathol. Exp. Neurol..

[50] Ferrer-Sueta G., Campolo N., Trujillo M., Bartesaghi S., Carballal S., Romero N., Alvarez B., Radi R. (2018). Biochemistry of Peroxynitrite and Protein Tyrosine Nitration. Chem. Rev..

[51] Szabo C., Ischiropoulos H., Radi R. (2007). Peroxynitrite: Biochemistry, pathophysiology and development of therapeutics. Nat. Rev. Drug Discov..

[52] Zinellu A., Tommasi S., Carru C., Sotgia S., Mangoni A.A. (2024). A systematic review and meta-analysis of nitric oxide-associated arginine metabolites in schizophrenia. Transl. Psychiatry.

[53] Janickova L., Schwaller B. (2020). Parvalbumin-Deficiency Accelerates the Age-Dependent ROS Production in Pvalb Neurons in vivo: Link to Neurodevelopmental Disorders. Front. Cell. Neurosci..

[54] Miyazaki I., Murakami S., Torigoe N., Kitamura Y., Asanuma M. (2016). Neuroprotective effects of levetiracetam target xCT in astrocytes in parkinsonian mice. J. Neurochem..

[55] Shih A.Y., Erb H., Sun X., Toda S., Kalivas P.W., Murphy T.H. (2006). Cystine/glutamate exchange modulates glutathione supply for neuroprotection from oxidative stress and cell proliferation. J. Neurosci..

[56] Spaas J., van Veggel L., Schepers M., Tiane A., van Horssen J., Wilson D.M., Moya P.R., Piccart E., Hellings N., Eijnde B.O. (2021). Oxidative stress and impaired oligodendrocyte precursor cell differentiation in neurological disorders. Cell. Mol. Life Sci..

[57] Bernstein H.G., Nussbaumer M., Vasilevska V., Dobrowolny H., Nickl-Jockschat T., Guest P.C., Steiner J. (2025). Glial cell deficits are a key feature of schizophrenia: Implications for neuronal circuit maintenance and histological differentiation from classical neurodegeneration. Mol. Psychiatry.

[58] Schmitt A., Tatsch L., Vollhardt A., Schneider-Axmann T., Raabe F.J., Roell L., Heinsen H., Hof P.R., Falkai P., Schmitz C. (2022). Decreased Oligodendrocyte Number in Hippocampal Subfield CA4 in Schizophrenia: A Replication Study. Cells.

[59] Falkai P., Rossner M.J., Raabe F.J., Wagner E., Keeser D., Maurus I., Roell L., Chang E., Seitz-Holland J., Schulze T.G. (2023). Disturbed Oligodendroglial Maturation Causes Cognitive Dysfunction in Schizophrenia: A New Hypothesis. Schizophr. Bull..

[60] Imai H., Matsuoka M., Kumagai T., Sakamoto T., Koumura T. (2017). Lipid Peroxidation-Dependent Cell Death Regulated by GPx4 and Ferroptosis. Curr. Top. Microbiol. Immunol..

[61] Ursini F., Maiorino M. (2020). Lipid peroxidation and ferroptosis: The role of GSH and GPx4. Free Radic. Biol. Med..

[62] Yang W.S., Stockwell B.R. (2016). Ferroptosis: Death by Lipid Peroxidation. Trends Cell Biol..

[63] Yang W.S., SriRamaratnam R., Welsch M.E., Shimada K., Skouta R., Viswanathan V.S., Cheah J.H., Clemons P.A., Shamji A.F., Clish C.B. (2014). Regulation of ferroptotic cancer cell death by GPX4. Cell.

[64] Zhou Z.D., Yi L.X., Wang D.Q., Lim T.M., Tan E.K. (2023). Role of dopamine in the pathophysiology of Parkinson’s disease. Transl. Neurodegener..

[65] Yang M., Wang C., Zhao G., Kong D., Liu L., Yuan S., Chen W., Feng C., Li Z. (2023). Comparative Analysis of the Pre- and Post-Medication Effects of Antipsychotic Agents on the Blood-Based Oxidative Stress Biomarkers in Patients with Schizophrenia: A Meta-Analysis. Curr. Neuropharmacol..

[66] Powell S.B., Sejnowski T.J., Behrens M.M. (2012). Behavioral and neurochemical consequences of cortical oxidative stress on parvalbumin-interneuron maturation in rodent models of schizophrenia. Neuropharmacology.

[67] Santos-Silva T., Lopes C.F.B., Ulgen D.H., Guimaraes D.A., Guimaraes F.S., Alberici L.C., Sandi C., Gomes F.V. (2025). Adolescent Stress-Induced Ventral Hippocampus Redox Dysregulation Underlies Behavioral Deficits and Excitatory/Inhibitory Imbalance Related to Schizophrenia. Schizophr. Bull..

[68] Raabe F.J., Slapakova L., Rossner M.J., Cantuti-Castelvetri L., Simons M., Falkai P.G., Schmitt A. (2019). Oligodendrocytes as A New Therapeutic Target in Schizophrenia: From Histopathological Findings to Neuron-Oligodendrocyte Interaction. Cells.

[69] Cassoli J.S., Guest P.C., Malchow B., Schmitt A., Falkai P., Martins-de-Souza D. (2015). Disturbed macro-connectivity in schizophrenia linked to oligodendrocyte dysfunction: From structural findings to molecules. npj Schizophr..

[70] Lin C.H., Lane H.Y. (2019). Early Identification and Intervention of Schizophrenia: Insight From Hypotheses of Glutamate Dysfunction and Oxidative Stress. Front. Psychiatry.

[71] Rambaud V., Marzo A., Chaumette B. (2022). Oxidative Stress and Emergence of Psychosis. Antioxidants.

[72] Emiliani F.E., Sedlak T.W., Sawa A. (2014). Oxidative stress and schizophrenia: Recent breakthroughs from an old story. Curr. Opin. Psychiatry.

[73] Jiang F., Jin T., Yang Q., Wang P., Ji L., Ma X., Zhang C., Tian Q., Zhang X. (2026). Abnormal plasma oxidative stress markers in first-episode schizophrenia and associations with clinical symptoms and cognitive function. Schizophrenia.

[74] Zhu Z., Wang Z., Yuan X., Zou Y., Zheng C., Wen Y., Hei G., Song X., Shi Y. (2026). Multimodal neuroimaging reveals brain neurochemical disturbances associated with superoxide dismutase in first-episode drug-naive schizophrenia. Transl. Psychiatry.

[75] Peng Z., Jia Q., Mao J., Jiang S., Ma Q., Luo X., An Z., Huang A., Ma C., Yi Q. (2025). The role of ferroptosis and oxidative stress in cognitive deficits among chronic schizophrenia patients: A multicenter investigation. Schizophrenia.

[76] Juchnowicz D., Dzikowski M., Rog J., Waszkiewicz N., Zalewska A., Maciejczyk M., Karakula-Juchnowicz H. (2021). Oxidative Stress Biomarkers as a Predictor of Stage Illness and Clinical Course of Schizophrenia. Front. Psychiatry.

[77] Zheng W., Zhang Q.E., Cai D.B., Yang X.H., Qiu Y., Ungvari G.S., Ng C.H., Berk M., Ning Y., Xiang Y. (2018). N-acetylcysteine for major mental disorders: A systematic review and meta-analysis of randomized controlled trials. Acta Psychiatr. Scand..

[78] Neill E., Rossell S.L., Yolland C., Meyer D., Galletly C., Harris A., Siskind D., Berk M., Bozaoglu K., Dark F. (2022). N-Acetylcysteine (NAC) in Schizophrenia Resistant to Clozapine: A Double-Blind, Randomized, Placebo-Controlled Trial Targeting Negative Symptoms. Schizophr. Bull..

[79] Zhang Q., Liu Z., Wang T., Yu M., Li X. (2024). Efficacy and acceptability of adjunctive n-acetylcysteine for psychotic disorders: Systematic review and meta-analysis. Hum. Psychopharmacol..

[80] Huang J., Chen A., Jin H., Liu F., Hei G., Teng Z., Xiao J., Wu R., Zhao J., Davis J.M. (2025). Efficacy and Safety of Sulforaphane Added to Antipsychotics for the Treatment of Negative Symptoms of Schizophrenia: A Randomized Controlled Trial. J. Clin. Psychiatry.

[81] Chen H., Lu J., Zou T., Teng Z., Qin Y., Wu R., Yan Y., Fu K., Jiang W., Ju Y. (2025). Effects of sulforaphane on negative symptoms and cognitive impairments in chronic schizophrenia patients: A randomized double-blind trial. J. Psychiatr. Res..

[82] Kassar O., Mansour M.E.M., Farag N., Selim A., Kewiaa Y., Yousef O., Hassan O. (2025). Efficacy and safety of sulforaphane in schizophrenia: A systematic review and meta-analysis of randomized controlled trials. BMC Psychiatry.

[83] Pawelczyk T., Grancow-Grabka M., Trafalska E., Szemraj J., Pawelczyk A. (2017). Oxidative stress reduction related to the efficacy of n-3 polyunsaturated fatty acids in first episode schizophrenia: Secondary outcome analysis of the OFFER randomized trial. Prostaglandins Leukot. Essent. Fat. Acids.

[84] Bentsen H., Osnes K., Refsum H., Solberg D.K., Bohmer T. (2013). A randomized placebo-controlled trial of an omega-3 fatty acid and vitamins E+C in schizophrenia. Transl. Psychiatry.

[85] Bentsen H., Landro N.I. (2018). Neurocognitive effects of an omega-3 fatty acid and vitamins E+C in schizophrenia: A randomised controlled trial. Prostaglandins Leukot. Essent. Fat. Acids.

[86] Stedehouder J., Kushner S.A. (2017). Myelination of parvalbumin interneurons: A parsimonious locus of pathophysiological convergence in schizophrenia. Mol. Psychiatry.

[87] Klauser P., Xin L., Fournier M., Griffa A., Cleusix M., Jenni R., Cuenod M., Gruetter R., Hagmann P., Conus P. (2018). N-acetylcysteine add-on treatment leads to an improvement of fornix white matter integrity in early psychosis: A double-blind randomized placebo-controlled trial. Transl. Psychiatry.

[88] Gray N.E., Farina M., Tucci P., Saso L. (2022). The Role of the NRF2 Pathway in Maintaining and Improving Cognitive Function. Biomedicines.

[89] Patel S., Sharma D., Kalia K., Tiwari V. (2017). Crosstalk between endoplasmic reticulum stress and oxidative stress in schizophrenia: The dawn of new therapeutic approaches. Neurosci. Biobehav. Rev..

[90] Wang X., Pinto-Duarte A., Sejnowski T.J., Behrens M.M. (2013). How Nox2-containing NADPH oxidase affects cortical circuits in the NMDA receptor antagonist model of schizophrenia. Antioxid. Redox Signal..

[91] Ermakov E.A., Dmitrieva E.M., Parshukova D.A., Kazantseva D.V., Vasilieva A.R., Smirnova L.P. (2021). Oxidative Stress-Related Mechanisms in Schizophrenia Pathogenesis and New Treatment Perspectives. Oxidative Med. Cell. Longev..

[92] Liang J.Q., Chen X., Cheng Y. (2022). Paeoniflorin Rescued MK-801-Induced Schizophrenia-Like Behaviors in Mice via Oxidative Stress Pathway. Front. Nutr..

[93] Dwamena A., Asadi Y., Gilstrap E., Wang H. (2025). Proteasomal dysfunction in the mouse forebrain induces mitochondrial DNA release, cGAS-STING signaling activation, and necroptosis. J. Neuropathol. Exp. Neurol..

[94] Brand B.A., de Boer J.N., Sommer I.E.C. (2021). Estrogens in schizophrenia: Progress, current challenges and opportunities. Curr. Opin. Psychiatry.

